# Supplementary material for: Measuring remote working skills: Scale development and validation study
Source: PLoS One. 2024 Apr 11;19(4):e0299074. doi: 10.1371/journal.pone.0299074 (PMC11008841; doi:10.1371/journal.pone.0299074)
Supplement: S1 File — (DOCX) [file pone.0299074.s001.docx]

| Remote Working Skills Scale | | | | | | | |
| --- | --- | --- | --- | --- | --- | --- | --- |
| Item | **Rate the items regarding your remote working experience from 1 to 7 (Strongly disagree to strongly agree)** | | | | | | |
| Cybersecurity skills | | | | | | | |
| I can protect digital decives physically. | **1** | **2** | **3** | **4** | **5** | **6** | **7** |
| I can implement the security strategy recommended by the institution. | **1** | **2** | **3** | **4** | **5** | **6** | **7** |
| I can provide digital privacy of mine and others. | **1** | **2** | **3** | **4** | **5** | **6** | **7** |
| I can protect the digital device from external threats such as viruses etc. | **1** | **2** | **3** | **4** | **5** | **6** | **7** |
| I can provide data privacy. | **1** | **2** | **3** | **4** | **5** | **6** | **7** |
| Problem solving skills | | | | | | | |
| I can choose the appropriate tool and method to solve problems (technical and non-technical). | **1** | **2** | **3** | **4** | **5** | **6** | **7** |
| I can solve problems that arise when the current technologies do not work. | **1** | **2** | **3** | **4** | **5** | **6** | **7** |
| I can select appropriate technology (tool, device, application, software, service, etc.) required by the task. | **1** | **2** | **3** | **4** | **5** | **6** | **7** |
| I can choose a tool that fits the purpose and evaluate the tool’s effectiveness. | **1** | **2** | **3** | **4** | **5** | **6** | **7** |
| I can use new technological devices (new software, interfaces, hardware, etc.). | **1** | **2** | **3** | **4** | **5** | **6** | **7** |
| I can learn to do something new with current technologies. | **1** | **2** | **3** | **4** | **5** | **6** | **7** |
| I can keep digital competencies up to date. | **1** | **2** | **3** | **4** | **5** | **6** | **7** |
| I can manage efficiently the excessive flow of information brought by information and communication technologies. | **1** | **2** | **3** | **4** | **5** | **6** | **7** |
| Time management skills | | | | | | | |
| I can calculate accurately how long the tasks will take. | **1** | **2** | **3** | **4** | **5** | **6** | **7** |
| I can sort tasks by importance and degree of urgency. | **1** | **2** | **3** | **4** | **5** | **6** | **7** |
| I can use the time worked online (working hours defined by the employer) effectively. | **1** | **2** | **3** | **4** | **5** | **6** | **7** |
| I can maintain work-life balance. | **1** | **2** | **3** | **4** | **5** | **6** | **7** |
| I can cope with distractions at home (demands of family members, needs of pets, housework, noise, etc.). | **1** | **2** | **3** | **4** | **5** | **6** | **7** |
| Verbal communication skills | | | | | | | |
| I can participate in the online conversations and dialogues. | **1** | **2** | **3** | **4** | **5** | **6** | **7** |
| I can resolve the conflicts in online environments. | **1** | **2** | **3** | **4** | **5** | **6** | **7** |
| I can receive feedback. | **1** | **2** | **3** | **4** | **5** | **6** | **7** |
| I can give feedback. | **1** | **2** | **3** | **4** | **5** | **6** | **7** |
| I can communicate correctly in times of crises. | **1** | **2** | **3** | **4** | **5** | **6** | **7** |
| I can focus on the main points (avoiding unnecessary details, keeping words, being simple, etc.). | **1** | **2** | **3** | **4** | **5** | **6** | **7** |
| I can listen actively (asking questions, trying to understand, focusing, etc.). | **1** | **2** | **3** | **4** | **5** | **6** | **7** |
| Written communication skills | | | | | | | |
| I can spell the words correctly. | **1** | **2** | **3** | **4** | **5** | **6** | **7** |
| I can use the grammar correctly. | **1** | **2** | **3** | **4** | **5** | **6** | **7** |
| I can use a simple, easy, understandable and fluent language. | **1** | **2** | **3** | **4** | **5** | **6** | **7** |
| I can express ideas clearly. | **1** | **2** | **3** | **4** | **5** | **6** | **7** |
| I can write in a way that the reader can understand. | **1** | **2** | **3** | **4** | **5** | **6** | **7** |
| I can transmit information accurately (to the right person, at the right time, with the right tool, etc.). | **1** | **2** | **3** | **4** | **5** | **6** | **7** |
| I can write in an appropriate format for different readers (employees, customers, suppliers, public institutions, etc.). | **1** | **2** | **3** | **4** | **5** | **6** | **7** |
| I can use verified information from different resources to ensure the accuracy of the content. | **1** | **2** | **3** | **4** | **5** | **6** | **7** |
| I can use a professional writing style. | **1** | **2** | **3** | **4** | **5** | **6** | **7** |
| I can write clear instructions. | **1** | **2** | **3** | **4** | **5** | **6** | **7** |
| I can express myself quickly and accurately while using instant messaging tools ( Whatsapp, Telegram, etc.). | **1** | **2** | **3** | **4** | **5** | **6** | **7** |
