# Supplementary material for: Measuring remote working skills: Scale development and validation study
Source: PLoS One. 2024 Apr 11;19(4):e0299074. doi: 10.1371/journal.pone.0299074 (PMC11008841; doi:10.1371/journal.pone.0299074)
Supplement: S1 Data — (ZIP) [file pone.0299074.s002.zip › Opinions of 10 experts/Expert 3.docx]

**Uzaktan Çalışma Becerileri Uzman Görüş Formu**

Sayın katılımcı,

Uzaktan çalışma becerilerine ilişkin ölçek geliştirme çalışması kapsamında, aşağıdaki tabloda yer alan ifadeleri “aynen kalsın”, “iptal edilsin” veya “şu şekilde değiştirilsin” olarak değerlendirmenizi talep etmekteyiz. “Aynen kalsın” veya “iptal edilsin” görüşünüz için ilgili kutucuğa “X” işareti koymanız yeterlidir. Ancak ifadenin değiştirilmesini istiyorsanız, lütfen önerdiğiniz halini “şu şekilde değiştirilsin” kutucuğu altına yazınız. Ayrıca önerilen ifadeler dışında yeni bir ifade eklemek isterseniz, her boyutun altında yer alan “Diğer 1”, Diğer 2” ve “Diğer 3” satırlarını kullanabilirsiniz. Değerli görüşleriniz ve katılımınız için teşekkür ederiz.

İletişim: [sbenligi@anadolu.edu.tr](mailto:sbenligi@anadolu.edu.tr)

|  | **İFADELER** | **Aynen kalsın** | **İptal edilsin** | **Şu şekilde** değiştirilsin |
| --- | --- | --- | --- | --- |
| **Güvenlik Boyutu** | Dijital cihazları korumak. | X |  |  |
|  | Güvenlik stratejisi geliştirmek ve düzenli olarak güncellemek. |  |  | Önerilen güvenlik stratejilerini uygulamak. |
|  | Kendisinin ve diğerlerinin çevrimiçi gizliliğini korumak. | X |  |  |
|  | Cihaz tehdit altındayken (virüs vb.) çözüm geliştirmek. |  | X |  |
|  | Siber zorbalıktan korunmak. |  | X |  |
|  | Veri gizliliğini sağlamak. | X |  |  |
|  | Teknoloji kullanımından kaynaklı sağlık sorunlarından (ergonomik, psikolojik vs.) kaçınmak. | X |  |  |
|  | Enerji tasarrufu için önlem almak. | X |  |  |
|  | Çevrimiçi ve çevrimdışı dünyalar arasında denge kurmak. | X |  |  |
|  | Diğer 1: Kurumun itibarına zarar verecek paylaşımlardan kaçınmak. |  |  |  |
|  | Diğer 2: |  |  |  |
|  | Diğer 3: |  |  |  |
|  |  |  |  |  |
| **Problem Çözme Boyutu** | Teknolojiler işe yaramadığında ortaya çıkan sorunları çözmek. | X |  |  |
|  | Sıra dışı görev için uygun araç, cihaz, uygulama, yazılım veya hizmet seçmek. | X |  |  |
|  | Teknolojik fırsatları keşfederek rutin olmayan bir görevi yerine getirmek. |  | X | (Üsttekinin tekrarı) |
|  | Teknolojileri yaratıcı bir şekilde kullanmak. |  | X | (İş araçları amacı dışında kullanılmamalı) |
|  | Amaca uygun bir araç seçmek ve aracın etkililiğini değerlendirmek. | X |  |  |
|  | Yeni teknolojik araçları kullanmak. |  |  | Yeni teknolojik araçları kullanma becerisi kazanmak. |
|  | Teknik ve teknik olmayan sorunları çözmek için doğru araç ve yönetimi seçmek. |  | X | (Belirsiz) |
|  | Yenilikçi ve yaratıcı çıktıların üretilmesinde başkalarıyla işbirliği yapmak. | X |  |  |
|  | Teknolojilerle yeni bir şeyler yapmayı öğrenmek. |  | X |  |
|  | Dijital yetkinlik ihtiyaçlarını sürekli güncellemek. | X |  |  |
|  | Teknolojiler işe yaramadığında ortaya çıkan sorunları çözmek. |  | X | (Tekrarlanmış) |
|  | Diğer 1: Kullanılan cihaz, yazılım ve hizmetlerin daha düşük maliyetli alternatiflerini tespit etmek. |  |  |  |
|  | Diğer 2: |  |  |  |
|  | Diğer 3: |  |  |  |
|  |  |  |  |  |
| **Zaman Yönetimi Boyutu** | Ne yapılması gerektiğini net olarak anlamak. |  |  | Görev ve sorumlulukları net olarak anlamak. |
|  | Görevlerin ne kadar zaman alacağını doğru hesaplamak. | X |  |  |
|  | Görevleri önem derecesine göre sıralamak. |  |  | Görevleri önem derecesine göre doğru olarak sıralamak. |
|  | Zaman tüketici şeylerden korunmak |  |  | Zamanı verimli kullanmak. |
|  | Diğer 1: İş-hayat dengesini korumak |  |  |  |
|  | Diğer 2: |  |  |  |
|  | Diğer 3: |  |  |  |
|  |  |  |  |  |
| **Sözlü İletişim Boyutu** | Uygun gramer kullanmak. |  |  | Uygun dilbilgisi kullanmak. |
|  | Diyaloğa dâhil olmak. | X |  |  |
|  | İnisiyatif almak. | X |  |  |
|  | İkna edici olmak. | X |  |  |
|  | Çatışmayı çözmek. |  |  | Çatışmayı çözümlemek. |
|  | Toplantı için planlama yapmak. | X |  |  |
|  | Toplantıya katılmak. |  |  | Toplantıya zamanında katılmak. |
|  | Kötü haberi en uygun şekilde vermek. | X |  |  |
|  | Telefonu etkili bir şekilde kullanmak. | X |  |  |
|  | Geri bildirim almak. | X |  |  |
|  | Kriz anında doğru iletişim kurmak. | X |  |  |
|  | Geri bildirim vermek. | X |  |  |
|  | Takım iletişimi kurmak. | X |  |  |
|  | Diğer 1: |  |  |  |
|  | Diğer 2: |  |  |  |
|  | Diğer 3: |  |  |  |
|  |  |  |  |  |
| **Yazılı İletişim Boyutu** | Kelimeleri doğru yazmak. |  |  | Yazım hatası yapmamak. |
|  | Noktalama işaretlerini doğru kullanmak. | X |  |  |
|  | Grameri doğru kullanmak. |  |  | Dilbilgisi kurallarına uygun yazmak. |
|  | Fikirleri açık bir şekilde ifade etmek. | X |  |  |
|  | İşletmede çalışanların anlayacağı bir formatta yazmak. |  |  | Okuyucunun anlayacağı bir biçimde yazmak. |
|  | İkna edici yazmak. |  |  | İkna edici bir şekilde yazmak. |
|  | Bilgiyi doğru bir şekilde iletmek. | X |  |  |
|  | Farklı okuyucular (müşteriler, çalışanlar, kamu kurumları vb.) için uygun formatta yazmak. |  |  | (Tekrar) |
|  | Mantıklı bir şekilde yazmak. |  |  | Mantık hataları ve safsatalardan kaçınmak. |
|  | İlgili bilgiyi farklı kaynaklardan toplamak. |  |  | Kullanılan bilgileri birden fazla kaynaktan teyit etmek. |
|  | Farklı kaynaklardan elde edilen bilgiyi özetlemek ve açık bir şekilde iletmek. | X |  |  |
|  | Profesyonel bir yazım biçimi kullanmak. |  |  | Profesyonel bir üslup kullanmak. |
|  | Açık yönergeler yazmak. | X |  |  |
|  | Diğer 1: |  |  |  |
|  | Diğer 2: |  |  |  |
|  | Diğer 3: |  |  |  |
