# Supplementary material for: Measuring remote working skills: Scale development and validation study
Source: PLoS One. 2024 Apr 11;19(4):e0299074. doi: 10.1371/journal.pone.0299074 (PMC11008841; doi:10.1371/journal.pone.0299074)
Supplement: S1 Data — (ZIP) [file pone.0299074.s002.zip › Opinions of 10 experts/Expert 4.docx]

**Uzaktan Çalışma Becerileri Uzman Görüş Formu**

Sayın katılımcı,

Uzaktan çalışma becerilerine ilişkin ölçek geliştirme çalışması kapsamında, aşağıdaki tabloda yer alan ifadeleri “aynen kalsın”, “iptal edilsin” veya “şu şekilde değiştirilsin” olarak değerlendirmenizi talep etmekteyiz. “Aynen kalsın” veya “iptal edilsin” görüşünüz için ilgili kutucuğa “X” işareti koymanız yeterlidir. Ancak ifadenin değiştirilmesini istiyorsanız, lütfen önerdiğiniz halini “şu şekilde değiştirilsin” kutucuğu altına yazınız. Ayrıca önerilen ifadeler dışında yeni bir ifade eklemek isterseniz, her boyutun altında yer alan “Diğer 1”, Diğer 2” ve “Diğer 3” satırlarını kullanabilirsiniz. Değerli görüşleriniz ve katılımınız için teşekkür ederiz.

İletişim: [sbenligi@anadolu.edu.tr](mailto:sbenligi@anadolu.edu.tr)

|  | **İFADELER** | **Aynen kalsın** | **İptal edilsin** | **Şu şekilde** değiştirilsin |
| --- | --- | --- | --- | --- |
| **Güvenlik Boyutu** | Dijital cihazları korumak. |  |  | Neye karşı korumak, dijital cihazları ……korumak |
|  | Güvenlik stratejisi geliştirmek ve düzenli olarak güncellemek. |  | * |  |
|  | Kendisinin ve diğerlerinin çevrimiçi gizliliğini korumak. |  | * | 2. sonraki soru ile aynı gibi |
|  | Cihaz tehdit altındayken (virüs vb.) çözüm geliştirmek. |  |  | Cihaza dışarıdan gelebilecek virüs vb tehditler için çözüm geliştirebilmek |
|  | Siber zorbalıktan korunmak. |  |  | Alay etme, küfür, küçük düşürme vb. siber zorbalıkları yönetebilme |
|  | Veri gizliliğini sağlamak. |  |  | Kişisel verilerimin gizliliğini sağlayabilmek |
|  | Teknoloji kullanımından kaynaklı sağlık sorunlarından (ergonomik, psikolojik vs.) kaçınmak. |  |  | Teknoloji kullanımından kaynaklı sağlık sorunlarıyla başedebilmek (göz, boyun problemleri vs.) |
|  | Enerji tasarrufu için önlem almak. |  |  | Teknolojiyi tasarruflu kullanabilmek |
|  | Çevrimiçi ve çevrimdışı dünyalar arasında denge kurmak. |  |  | Çevrimiçi ve çevrimdışı dünya arasındaki dengeyi kurabilmek |
|  | Diğer 1: |  |  | Bir üstteki soru ile ilgili tek bir genel soru yerine birkaç farklı ifade oluşturulabilir. Ev işleri, çocuk, kapı çalması, dikkati dağıtabilecek ve öncelikleri belirlemeyi zorlaştıracak durumlar hakkında |
|  | Diğer 2: |  |  |  |
|  | Diğer 3: |  |  |  |
|  |  |  |  |  |
| **Problem Çözme Boyutu** | Teknolojiler işe yaramadığında ortaya çıkan sorunları çözmek. |  |  | Teknoloji ile ilgili sorun çıktığında çözebilmek |
|  | Sıra dışı görev için uygun araç, cihaz, uygulama, yazılım veya hizmet seçmek. |  |  | Uygun teknolojiyi seçebilmek |
|  | Teknolojik fırsatları keşfederek rutin olmayan bir görevi yerine getirmek. |  | * |  |
|  | Teknolojileri yaratıcı bir şekilde kullanmak. |  | * |  |
|  | Amaca uygun bir araç seçmek ve aracın etkililiğini değerlendirmek. |  |  | İki ayrı soru, ayrı ayrı sorulabilir |
|  | Yeni teknolojik araçları kullanmak. |  | * | Yeni yazılımlar, ara yüzler gibi açıklamalar yapılabilir |
|  | Teknik ve teknik olmayan sorunları çözmek için doğru araç ve yönetimi seçmek. |  |  | Teknik ve teknik olmayan ayrı sorulabilir ve örneklendirilebilir |
|  | Yenilikçi ve yaratıcı çıktıların üretilmesinde başkalarıyla işbirliği yapmak. | * |  |  |
|  | Teknolojilerle yeni bir şeyler yapmayı öğrenmek. | * |  |  |
|  | Dijital yetkinlik ihtiyaçlarını sürekli güncellemek. |  |  | Daha açık bir ifade olabilir |
|  | Teknolojiler işe yaramadığında ortaya çıkan sorunları çözmek. | * |  |  |
|  | Diğer 1: |  |  |  |
|  | Diğer 2: |  |  |  |
|  | Diğer 3: |  |  |  |
|  |  |  |  |  |
| **Zaman Yönetimi Boyutu** | Ne yapılması gerektiğini net olarak anlamak. | * |  |  |
|  | Görevlerin ne kadar zaman alacağını doğru hesaplamak. | * |  |  |
|  | Görevleri önem derecesine göre sıralamak. |  |  | Görevlerin önem derecesini belirleyebilmek |
|  | Zaman tüketici şeylerden korunmak |  |  | Online çalışılan zamanı etkin kullanabilmek |
|  | Diğer 1: |  |  |  |
|  | Diğer 2: |  |  |  |
|  | Diğer 3: |  |  |  |
|  |  |  |  |  |
| **Sözlü İletişim Boyutu** | Uygun gramer kullanmak. |  |  | Yazı dilini doğru kullanmak |
|  | Diyaloğa dâhil olmak. |  |  | Konuşma ve diyaloglara katılabilmek |
|  | İnisiyatif almak. | * |  |  |
|  | İkna edici olmak. | * |  |  |
|  | Çatışmayı çözmek. |  |  | Online iş ortamlarındaki çatışmaları yönetebilmek |
|  | Toplantı için planlama yapmak. | * |  |  |
|  | Toplantıya katılmak. |  |  | Toplantılara katılmak |
|  | Kötü haberi en uygun şekilde vermek. |  | * |  |
|  | Telefonu etkili bir şekilde kullanmak. | * |  |  |
|  | Geri bildirim almak. | * |  |  |
|  | Kriz anında doğru iletişim kurmak. | * |  |  |
|  | Geri bildirim vermek. | * |  |  |
|  | Takım iletişimi kurmak. |  | * | Açık değil |
|  | Diğer 1: |  |  | Gereken konulara odaklanmak(gereksiz konuşmalar yapmamak, uzatmamak, sadede gelebilmek gibi) |
|  | Diğer 2: |  |  |  |
|  | Diğer 3: |  |  |  |
|  |  |  |  |  |
| **Yazılı İletişim Boyutu** | Kelimeleri doğru yazmak. |  |  | İlk 3 soru tek bir ifade ile yazılabilir |
|  | Noktalama işaretlerini doğru kullanmak. |  |  |  |
|  | Grameri doğru kullanmak. |  |  |  |
|  | Fikirleri açık bir şekilde ifade etmek. | * |  |  |
|  | İşletmede çalışanların anlayacağı bir formatta yazmak. |  | * |  |
|  | İkna edici yazmak. |  | * |  |
|  | Bilgiyi doğru bir şekilde iletmek. | * |  |  |
|  | Farklı okuyucular (müşteriler, çalışanlar, kamu kurumları vb.) için uygun formatta yazmak. |  |  | Herkes tarafından anlaşılabilecek bir şekilde yazmak |
|  | Mantıklı bir şekilde yazmak. |  | * |  |
|  | İlgili bilgiyi farklı kaynaklardan toplamak. |  |  | Gerektiğinde farklı kaynaklardan bilgi toplayabilmek |
|  | Farklı kaynaklardan elde edilen bilgiyi özetlemek ve açık bir şekilde iletmek. |  |  | ? |
|  | Profesyonel bir yazım biçimi kullanmak. | * |  |  |
|  | Açık yönergeler yazmak. | * |  |  |
|  | Diğer 1: |  |  | Etkili sunum materyalleri geliştirmek |
|  | Diğer 2: |  |  |  |
|  | Diğer 3: |  |  |  |
