# Supplementary material for: Measuring remote working skills: Scale development and validation study
Source: PLoS One. 2024 Apr 11;19(4):e0299074. doi: 10.1371/journal.pone.0299074 (PMC11008841; doi:10.1371/journal.pone.0299074)
Supplement: S1 Data — (ZIP) [file pone.0299074.s002.zip › Opinions of 10 experts/Expert 6.docx]

**Uzaktan Çalışma Becerileri Uzman Görüş Formu**

Sayın katılımcı,

Uzaktan çalışma becerilerine ilişkin ölçek geliştirme çalışması kapsamında, aşağıdaki tabloda yer alan ifadeleri “aynen kalsın”, “iptal edilsin” veya “şu şekilde değiştirilsin” olarak değerlendirmenizi talep etmekteyiz. “Aynen kalsın” veya “iptal edilsin” görüşünüz için ilgili kutucuğa “X” işareti koymanız yeterlidir. Ancak ifadenin değiştirilmesini istiyorsanız, lütfen önerdiğiniz halini “şu şekilde değiştirilsin” kutucuğu altına yazınız. Ayrıca önerilen ifadeler dışında yeni bir ifade eklemek isterseniz, her boyutun altında yer alan “Diğer 1”, Diğer 2” ve “Diğer 3” satırlarını kullanabilirsiniz. Değerli görüşleriniz ve katılımınız için teşekkür ederiz.

İletişim: [sbenligi@anadolu.edu.tr](mailto:sbenligi@anadolu.edu.tr)

|  | **İFADELER** | **Aynen kalsın** | **İptal edilsin** | **Şu şekilde** değiştirilsin |
| --- | --- | --- | --- | --- |
| **Güvenlik Boyutu** | Dijital cihazları korumak. | X |  |  |
|  | Güvenlik stratejisi geliştirmek ve düzenli olarak güncellemek. | X |  |  |
|  | Kendisinin ve diğerlerinin çevrimiçi gizliliğini korumak. | X |  |  |
|  | Cihaz tehdit altındayken (virüs vb.) çözüm geliştirmek. | X |  |  |
|  | Siber zorbalıktan korunmak. | X |  |  |
|  | Veri gizliliğini sağlamak. | X |  |  |
|  | Teknoloji kullanımından kaynaklı sağlık sorunlarından (ergonomik, psikolojik vs.) kaçınmak. |  |  | Calisma ortaminin ergonomik olmasi icin maddi ve manevi destegin is sagligi ve guvenligi departmani tarafindan saglanmasi. |
|  | Enerji tasarrufu için önlem almak. | X |  |  |
|  | Çevrimiçi ve çevrimdışı dünyalar arasında denge kurmak. | X |  |  |
|  | Diğer 1: |  |  |  |
|  | Diğer 2: |  |  |  |
|  | Diğer 3: |  |  |  |
|  |  |  |  |  |
| **Problem Çözme Boyutu** | Teknolojiler işe yaramadığında ortaya çıkan sorunları çözmek. | X |  |  |
|  | Sıra dışı görev için uygun araç, cihaz, uygulama, yazılım veya hizmet seçmek. | X |  |  |
|  | Teknolojik fırsatları keşfederek rutin olmayan bir görevi yerine getirmek. | X |  |  |
|  | Teknolojileri yaratıcı bir şekilde kullanmak. | X |  |  |
|  | Amaca uygun bir araç seçmek ve aracın etkililiğini değerlendirmek. |  | X |  |
|  | Yeni teknolojik araçları kullanmak. | X |  |  |
|  | Teknik ve teknik olmayan sorunları çözmek için doğru araç ve yönetimi seçmek. |  |  |  |
|  | Yenilikçi ve yaratıcı çıktıların üretilmesinde başkalarıyla işbirliği yapmak. | X |  |  |
|  | Teknolojilerle yeni bir şeyler yapmayı öğrenmek. | X |  |  |
|  | Dijital yetkinlik ihtiyaçlarını sürekli güncellemek. | X |  |  |
|  | Teknolojiler işe yaramadığında ortaya çıkan sorunları çözmek. | X |  |  |
|  | Diğer 1: |  |  |  |
|  | Diğer 2: |  |  |  |
|  | Diğer 3: |  |  |  |
|  |  |  |  |  |
| **Zaman Yönetimi Boyutu** | Ne yapılması gerektiğini net olarak anlamak. | X |  |  |
|  | Görevlerin ne kadar zaman alacağını doğru hesaplamak. |  | X |  |
|  | Görevleri önem derecesine göre sıralamak. | X |  |  |
|  | Zaman tüketici şeylerden korunmak |  | X |  |
|  | Diğer 1: |  |  |  |
|  | Diğer 2: |  |  |  |
|  | Diğer 3: |  |  |  |
|  |  |  |  |  |
| **Sözlü İletişim Boyutu** | Uygun gramer kullanmak. |  | X |  |
|  | Diyaloğa dâhil olmak. |  | X |  |
|  | İnisiyatif almak. | X |  |  |
|  | İkna edici olmak. |  | X |  |
|  | Çatışmayı çözmek. | X |  |  |
|  | Toplantı için planlama yapmak. | X |  |  |
|  | Toplantıya katılmak. | X |  |  |
|  | Kötü haberi en uygun şekilde vermek. | X |  |  |
|  | Telefonu etkili bir şekilde kullanmak. | X |  |  |
|  | Geri bildirim almak. | X |  |  |
|  | Kriz anında doğru iletişim kurmak. | X |  |  |
|  | Geri bildirim vermek. | X |  |  |
|  | Takım iletişimi kurmak. | X |  |  |
|  | Diğer 1: |  |  |  |
|  | Diğer 2: |  |  |  |
|  | Diğer 3: |  |  |  |
|  |  |  |  |  |
| **Yazılı İletişim Boyutu** | Kelimeleri doğru yazmak. |  | X |  |
|  | Noktalama işaretlerini doğru kullanmak. |  | X |  |
|  | Grameri doğru kullanmak. |  | X |  |
|  | Fikirleri açık bir şekilde ifade etmek. | X |  |  |
|  | İşletmede çalışanların anlayacağı bir formatta yazmak. | X |  |  |
|  | İkna edici yazmak. |  | X |  |
|  | Bilgiyi doğru bir şekilde iletmek. | X |  |  |
|  | Farklı okuyucular (müşteriler, çalışanlar, kamu kurumları vb.) için uygun formatta yazmak. | X |  |  |
|  | Mantıklı bir şekilde yazmak. | X |  |  |
|  | İlgili bilgiyi farklı kaynaklardan toplamak. | X |  |  |
|  | Farklı kaynaklardan elde edilen bilgiyi özetlemek ve açık bir şekilde iletmek. | X |  |  |
|  | Profesyonel bir yazım biçimi kullanmak. |  | X |  |
|  | Açık yönergeler yazmak. | X |  |  |
|  | Diğer 1: |  |  |  |
|  | Diğer 2: |  |  |  |
|  | Diğer 3: |  |  |  |
